# Factors and outcomes in Severe Fever with Thrombocytopenia Syndrome (SFTS): A systematic review

**DOI:** 10.1016/j.amsu.2021.102501

**Published:** 2021-06-11

**Authors:** Herwati Dualis, Abraham Chin Zefong, Lim Kai Joo, Narinderjeet Kaur Dadar Singh, Syed Sharizman Syed Abdul Rahim, Richard Avoi, Mohammad Saffree Jeffree, Mohd Rohaizat Hassan, Mohd Yusof Ibrahim, Azizan Omar

**Affiliations:** a Department of Community and Family Medicine, Faculty of Medicine and Health Sciences, Universiti Malaysia Sabah, Jalan UMS, 88400, Kota Kinabalu, Sabah, Malaysia; b Department of Community Health, National University of Malaysia Medical Center, 56000, Kuala Lumpur, Malaysia

**Keywords:** Severe fever with thrombocytopenia syndrome, SFTS, Bunyavirus, Phlebovirus

## Abstract

**Background:**

An emerging infectious zoonosis known as Severe Fever with Thrombocytopenia Syndrome (SFTS) is discovered mainly in Japan, South Korea and China. SFTS virus (SFTSV) which is recently recognised as bunyavirus is borne by ticks such as *Haemaphysalis longicornis*. It has the capabilities to spread as develop clusters and become a considerable public health threat as this virus could experience rapid evolution via gene mutation. Case fatality rate has been reported up to higher than 30%. The aim of this review is to determine the associated risk factors of SFTS and its outcome.

**Materials and methods:**

Literature search was conducted using online databases PubMed, ScienceDirect, and Scopus. A total of 517 records were identified from searches in PubMed, ScienceDirect, and Scopus. From the final exclusions, a total of 26 studies were included for final analysis.

**Results:**

Associated risk factors to getting SFTS infection include occupation, history of bite from a tick, biological susceptibility, and owning of domestic animal. Fatality rates apart from single case reports range from 15.1% to 50% and are contributed by various factors including delay in hospital admission, high viral load, older age group and presence of comorbid and complication.

**Conclusion:**

A seroprevalence study can be conducted amongst the high-risk occupation group such as farmers and agricultural workers, as well as testing cases where viral fever is suspected but available tests for other diseases turns out negative.

## Highlights

• SFTSV is a bunyavirus borne by ticks such as *Haemaphysalis longicornis*.
• Public health threat if progress into rapid evolution via gene mutation.
• Case fatality rate has been reported up to higher than 30%.
• SFTS infection risk factors include occupation, tick bite history, biological susceptibility, and owning domestic animal.
• Fatality rates ranges from 15.1% - 50%.
• Fatality associated with delay admission, viral load, older age and comorbidity.

## 1 Introduction

A recently recognised bunyavirus (also known as phlebovirus, from the Bunyaviridae family) known as SFTS virus (SFTSV) causes Severe Fever with Thrombocytopenia Syndrome (SFTS), that appears to be borne by ticks such as *Haemaphysalis longicornis.* This emerging infectious zoonosis has been reported in Asian countries such as South Korea China and Japan [1]. This virus has first been identified in rural China in 2009, with 5–14 days of incubation, headache, myalgia, lymphadenopathy, hemorrhagia and complications of the central nervous system, clinical symptoms, high fever. Thrombocytopenia and leukocytopenia will be part of the laboratory findings. The rate of fatalities in cases was reported to be as low as 6% and above 30%. Persons with serious disease can develop multi-organ failures about 5 days after the disease starts. In 2012, Heartland virus and another SFTSV-related phlebovirus were also identified in Japan and Korea; two patients in the United States were isolated [2]. Differential diagnosis of SFTS include haemorrhagic fever with renal syndrome, human granulocytic anaplasmosis, dengue fever, spotted fever group rickettsioses and leptospirosis [3].

In a seroprevalence study done in China involving 2547 farmers found SFTSV antibodies in 1.30% [4]. While a combination seroprevalence study of humans and animals conducted in Japan found that 0.14% healthy individual over 50 years old and 18.7% of domestic and wild animals to be seropositive for SFTSV antibodies [5].

The case fatality rate for SFTS in 2015 in both Japan were more than 30% [6]. In South Korea, overall case fatality rate was also noted to be at 32.6% in which most cases occurred during the month of May to October. This tick-borne haemorrhagic fever presented in 3 phases: fever, multi-organ impairment and convalescence with increased C-reactive protein (CRP), confusion and prolonged activated partial thromboplastin time (aPTT) which can cause mortality [7]. Due to high fatality rate, there is a need in learning more about the disease which may help in management of cases in the clinical settings.

SFTS has the capabilities to spread as develop clusters especially if the reservoir available in the environment. Clusters of cases have been found where the bunyavirus virus confirmed by molecular epidemiology methods to have strains that were very closely related in a human-to-human cluster [8]. SFTS has become a considerable threat to public health as this virus could experience rapid evolution via gene mutation. There is no specific treatment of SFTS and avoiding tick bites is the paramount measure to prevent the infection and transmission of SFTSV. There is no vaccine against SFTSV available to date. Hence, it is imperative that we learn about the associated risk factors and outcome to enhance our preparedness strategies [9].

The aims of this systematic review are to determine the associated risk factors of SFTS and its different outcomes.

## 2 Materials and methods

### 2.1 Criteria for considering studies for this review

Any study design will be included but would expect to be observational/case studies rather than randomised controlled trials. Exclusion include all non-primary literature, such as reviews, dissertations, theses, editorials, protocol studies and clinical guidelines. The type of population will be people with confirmed Severe Fever with Thrombocytopenia Syndrome (SFTS), both clinically and laboratory confirmation, of any age, gender and severity. Papers from all over the world will be examined. Types of outcome measures will be outcome such as alive/dead. Studies that do not report outcome will be excluded. The types of settings include primary care or hospital admissions or secondary care.

### 2.2 Search methods for identification of studies

The search was conducted electronically to search for suitable papers via PubMed, ScienceDirect, Scopus databases using predetermined methodologies according to Cochrane methods. No other resource was used for identification of studies. Search criteria used were Severe Fever with Thrombocytopenia Syndrome OR SFTS AND factors AND/OR complications AND/OR mortality.

### 2.3 Data collection and analysis

**Fig. 1:**
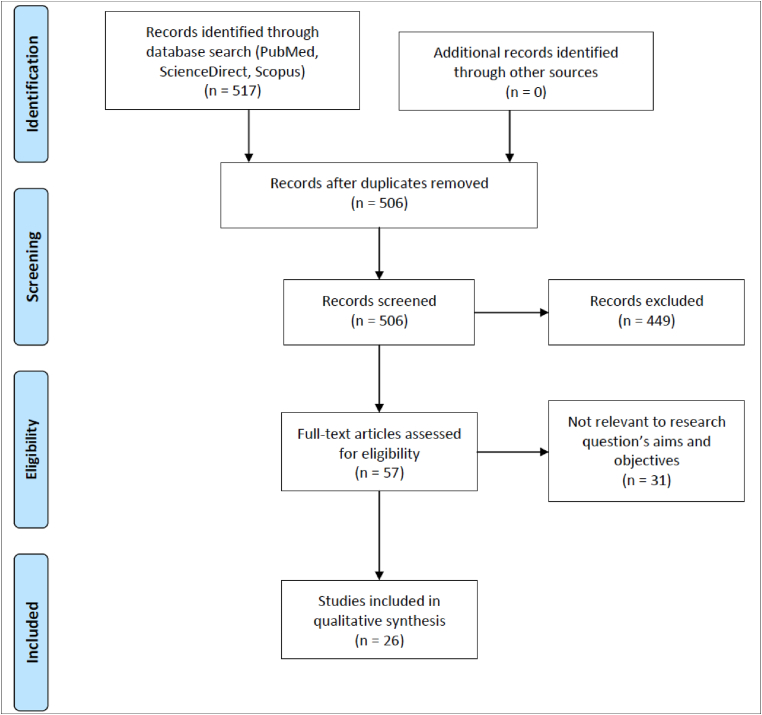
Prisma flow chart.

Records that matched the inclusion criteria were selected and those that matched the exclusion criteria were excluded. A PRISMA Flow Chart was done to identify included studies (Fig. 1) [10]. Data extraction form was produced in Excel format. Mendeley software was used as reference manager. Agreed data was extracted and using terminology clarified beforehand. First reviewer has reviewed, followed by second reviewer, which was done independently. There was not necessary for third reviewer to review as there was no disparity between the two initial reviews. As this is not comparison of intervention, hence no assessment of risk of bias was necessary. All data mined from the articles were presented in text and summary tables. The entire data extraction and synthesis process was carefully detailed, and objective third-party review (HD) was ready to be utilized if there was a need. AMSTAR 2 criteria were used to self-evaluate the systematic review and complied with majority of the criteria [11]. The review was also registered at Research Registry with unique identifying number (UIN) (reviewregistry1162) [12].

## 3 Results

By using the PICO framework, 517 records were identified from searches in PubMed, ScienceDirect, and Scopus. Out of these, there were 11 duplicates. A total of 506 records were screened and 449 excluded after review of title and abstract. A total of 57 full-text articles were assessed for eligibility. There was a total of 31 articles excluded due to non-relevant to research question's aims and objectives. In the end, 26 studies were included for qualitative analysis.

From the 26 studies, 12 studies we included for analysis of SFTS's associated risk factors (Table 1). Those studies were from China (10), Japan (1) and South Korea (1), ranging from the year 2013–2019. In addition, 14 studies were also included for the outcome analysis of SFTS (Table 2). Those study were from China (8) and Japan (6), ranging from 2012 to 2019.

**Table 1:** Characteristics of studies assessing the associated risk factors of SFTS.

Study	Method	Participants	Risk Factor	Measurement
Deng 2013 [13]	Cross-sectional	115 aged 17–89 years. Median age 55 years old	Occupation	99 (86.1%) of the 115 patients were farmers.
Pan 2013 [14]	Case Report	1 aged 40 years	Occupation	Collecting cotton, rice and tea
Ding 2014 [15]	Cross-sectional	71 aged 15–87 years. Median age 59	Biological	Age >40 (Incidence rate 7.1/100,100 vs 0.5/100,000 for those <40 years old)
			Occupation	All cases were farmers
Sun 2015 [16]	Case Report	3 aged 62–79 years old	Tick Bite	All had history of tick bite
			Biological	Genetic susceptibility as all were siblings
Sun 2016 [17]	Case Control	216 with median age 65 years old	Tick Bite	OR 6.592 (95% CI: 2.892–14.994)
			Breeding Domestic Animal	OR 1.745 (95% CI: 1.000–3.045)
Zhang 2017 [18]	Case Control	1020 SFTS patients and 1353 controls	Biological	PDGF-B rs1800818 polymorphism; OR 1.66 (95% CI: 1.28–2.16)
Sun 2017 [19]	Cross-sectional	5360 aged 40–80 years old	Occupation	Farmers (87.91%, 4712/5360)
Oh 2017 [20]	Retrospective Cohort	53 with mean age 65 years old	Occupation	Exposure to tick (38/53)
Kaneko 2017 [21]	Case Report	1 aged 56 years old	Occupation	Agricultural Worker
			Biological	Diabetes Mellitus
Jia 2018 [22]	Cross-sectional	83 with median age 59 years old	Occupation	78.30% were agricultural worker
Li 2018 [23]	Case Report	2 aged 66 and 79 years old	Tick Bite	Both had tick bites
			Biological	Elderly
Jung 2019 [24]	Retrospective Cohort	18 aged >60 years old	Tick Bite	12/18 had history of tick bite

**Table 2:** Characteristics of studies assessing the outcome of severe fever with thrombocytopenia syndrome.

Study	Method	Participants	Fatality	Contributing Factor
Choi 2016 [7]	Cross-sectional	74 non-fatal with median age 66, 46 fatal with median age 73.5	46/120	C-reactive protein that raised, prolonged activated partial thromboplastin times and confusion
Ding 2014 [15]	Case Report	9 aged 41–71 years old	2/9	Disseminated intravascular coagulation, ALT, AST, and LDH high, APTT elongated, high level CK, low fibrinogen
Zhang 2017 [18]	Cross-sectional	115 aged 28–91 years old	21/115	Lower total leukocytes, albumin (ALB) and platelet counts and higher serum alanine aspartate aminotransferase (AST), aminotransferase (ALT), γ-glutamyl transpeptidase (γ-GT), lactate dehydrogenase (LDH) and creatine kinase (CK) levels on admission
Jiao 2012 [25]	Cross-sectional	49 with mean age 54.4 years old	8/49	High blood viral RNA, higher figure of serum liver transaminase, more severe coagulation disorders, and elevated numbers of acute phase protein cytokines and chemokines
Cui 2014 [26]	Cross-sectional	357 aged 7–87 years old. Median age 61 years old	54/357	Decreased platelet counts at early stage, older age and increased AST level at middle stage, and decreased lymphocyte percentage and increased LDH level at late stage
Yoshikawa 2014 [27]	Cross-sectional	N/A	17/41	Viremia level
Cui 2015 [28]	Cohort	538 with mean age 59.5 ± 12.7 years old	87/538	Older age, longer delay between disease onset and hospital admission, pre-existing diabetes and myalgias, as well as the laboratory evaluations of higher virus load on admission, decreased WBC, PLT count, lymphocyte percentage and ALB, elevated neutrophils percentage, AST, ALT, LDH, CK, ALP, GGT, BUN and CREA.
Kaneyuki 2016 [29]	Case Report	1 aged 72 years old	1	Haemorrhagic manifestation
Yang 2016 [30]	Observational	148 with mean age 58.4	74/148	Higher viral loads were found to be significantly associated with severe disease outcome, older age and female gender
Chen 2018 [31]	Cross-sectional	4 aged 42–72 years old	4/4	SFTS-related invasive pulmonary aspergillosis
Kaneko 2018 [32]	Case Report	1 aged 53 years old	1	SFTS-related invasive pulmonary aspergillosis
Nakamura 2019 [33]	Case Report	1 aged 77 years old	1	High viral load and inflammatory cytokine
Zhang 2019 [34]	Cohort	2096 with mean age 61.4	340/2096	Comorbidities 779/2096 had at least 1 comorbidity
Miyamoto 2019 [35]	Case Report	1 aged 62 years old	0	Fulminant myocarditis

## 4 Discussion

Based on the 12 reviewed articles for associated risk factors to getting SFTS infection, they are categorised into four different categories, namely occupation, tick bite history, biological susceptibility, and owning of domestic or wild animals. Seven articles related it occupation mainly involved farmers and other agricultural workers. Four had history of exposure to tick bites, one article revealed risk from of owning cat or cattle. Another five articles were mainly on risk of age above 40 years old, PDGF-Brs 1800818 polymorphism and presence of other comorbid conditions.

Previous study has shown that exposure to goats, farming, and grazing might increase the risk of tick bites and infection with SFTSV in a healthy population [36]. Another study also demonstrated that the major risk factor was the tick bite two weeks before the onset of disease. In addition, the weeds and shrubs around the house lead to the fact that *Haemaphysalis longicornis* is classified as bush or brush ticks (transmission vehicle), which live freely in the area and await an appropriate host (e.g., small mammals, pets and wildlife) for this purpose [37]. The usage of gloves or boots, fastenings of the mouths of the pants and the shoes, the use of long-sleeved tops, and insect repellents during outdoor activities are protective factors and indicate that outdoor activities are a factor vulnerable to SFTSV infection [37]. Hilly areas were the main endemic areas [7].

Human-to-human transmission of SFTSV infection, including cases of transmission from patients to medical practitioners, have been reported [1,[38], [39], [40], [41]]. In these cases, transmission occurred through close contact with patient body fluids. This evidence indicates that strict contact precautions should be implemented to protect against nosocomial transmissions.

Based on 14 articles reviewed, several contributing factors towards fatal outcome of SFTS infection as well as several documented complications from SFTS infection could be identified. Among those factors were high viral load, older age, delay in hospital admission, presence of comorbid conditions, presence of SFTS-related complications and deranged blood profile such as coagulation, liver enzymes and cytokines. Fatality rate apart from single case report ranges from 15.1% to 50%, which is high [7,15,18,[25], [26], [27], [28], [29], [30], [31], [32], [33], [34], [35]].

Currently, it is common to misdiagnose SFTS and this pose a challenging situation for both to physicians and public health officials. There is a need to emphasis on improving SFTS diagnosis in poor-resource areas, for example rural areas in China, where no highly skilled laboratory or qualified laboratory technicians or high-cost PCR machines can provide viral laboratory validation for this diagnosis which leads to delay in diagnosis and appropriate treatment [42].

SFTS has characteristics of a rapid disease progression and high mortality; therefore, the early identification of critically ill patients is essential. Monitoring of blood parameters can give a picture of progression of disease and guide treatment protocol. There is no specific cure for SFTS infection, however, several measures have been described in literature with varying degree of success including oral ribavirin and plasma exchange [37]. Trial of new antiviral drug, favipiravir prove to be promising [37]. Supportive care, such as transfusion of fresh frozen plasma or platelet for hematologic abnormalities, methylprednisolone for acute lung injury or ARDS, albumin replacement for hypoalbuminemia, intravenous immunoglobulin for severe infection or encephalitis, granulocyte colony stimulating factor for leukopenia, and antibiotics for bacterial superinfection, would be the most important part of the treatment process.

Seroprevalence study can be conducted in high-risk areas such as hilly areas surrounded by shrubs and weeds, and amongst the high-risk occupation group such as farmers and agricultural workers. Testing can also be conducted for cases where viral fever is suspected but available tests are negative. Some potential limitations and potential biases in the review process include the lack of publication on SFTS despite searching in 3 major electronic databases, and also majority of articles were case reports, hence unable to determine causality or temporal relation as well as comparison with those without exposure.

As SFTS is an emerging disease, with climate change it's possible some vectors may influence the areas of habitat, survival, activity of ticks and the duration of the season when human are more exposed to ticks [43]. Thus in the future the possibility of tick-borne diseases may very well be increased hence the importance of this review for future prevention and case management.

## 5 Conclusion

There is no reported case of SFTS in some countries in Asia. However, as the risk factor described are almost similar to geographic and socioeconomic activity of the population, it is worth investing to prepare for surveillance of tick and human, more so due to the high fatality of SFTS infection. From this review, SFTS infection is associated with type of occupation, tick bite history, biological susceptibility, and owning of domestic animal. Fatality rates ranges from 15.1% to 50% and were influenced by factors such as delay in hospital admission, high viral load, older age group and presence of comorbid or complications.

## Availability of data and material

Data will be provided upon request.

## Registration of research studies

1. Name of the registry: (not involving human participants).

2. Unique Identifying number or registration ID: (not involving human participants).

3. Hyperlink to your specific registration (must be publicly accessible and will be checked): (not involving human participants).
